# Outcomes of Vitrectomy Under Air for Idiopathic Macular Hole

**DOI:** 10.4274/tjo.galenos.2019.89804

**Published:** 2019-12-31

**Authors:** Murat Karaçorlu, Mümin Hocaoğlu, Işıl Sayman Muslubaş, M. Giray Ersöz, Serra Arf

**Affiliations:** 1 İstanbul Retina Institute, Ophthalmology Clinic, İstanbul, Turkey

**Keywords:** Idiopathic macular hole, pars plana vitrectomy, vitrectomy under air

## Abstract

**Objectives:**

To evaluate the outcomes of 23-gauge pars plana vitrectomy (PPV) under air compared with standard PPV for idiopathic macular hole (MH).

**Materials and Methods:**

In this prospective, comparative, interventional case series, 42 eyes of 42 patients with idiopathic MH were enrolled. Twenty-one eyes had vitrectomy with an air-infused technique and 21 eyes underwent vitrectomy with a traditional balanced salt solution-infused technique as a control group. Effective vitrectomy time, total surgery time, microperimetry (MP1), and anatomical and functional results were evaluated.

**Results:**

The mean effective vitrectomy time was significantly lower in the air group than in the control group (7.5±0.3 min and 13.3±0.5 min, respectively, P<0.001). The mean total surgery time was significantly lower in the air group than the control group (21.8±2.0 min and 25.9±1.1 min, respectively, P<0.001). There were no statistically significant changes between preoperative and 3-month postoperative retinal sensitivity values evaluated by MP1 in either group. Anatomical success at 3 months was 100% in both groups. Intraoperative complications noted during the air-infused vitrectomy were retinal touch (10%) and sudden hypotony (10%); in the two pseudophakic eyes, migration of air into the anterior chamber occurred in one (50%) and fogging of the intraocular lens in one eye (50%).

**Conclusion:**

Vitrectomy under air infusion for idiopathic MH showed some advantages over a traditional vitrectomy technique in terms of vitreous visualization, effective vitrectomy time, and total surgery duration, without significantly increasing intraoperative and postoperative complication rates. Postoperative microperimetry results indicated no specific damage to the retina or optic nerve related to the continuous air infusion.

## Introduction

A full-thickness macular hole (MH) is defined as an anatomical defect in the fovea with interruption of all neural retinal layers from the internal limiting membrane (ILM) to the retinal pigment epithelium.^1^ An annual incidence of 7.4 per 100 000 inhabitants has been reported recently.^2^ The Gass classification of MH is based on careful clinical examination and divides MHs into four stages.^3^ Although this system is still quoted widely and adaptations of it are in clinical use, optical coherence tomography (OCT)-based anatomical data have added much to our understanding of the pathogenesis and progression of MH.^1,4^

Recently, MH surgery has greatly benefited from the advent of small-gauge transconjunctival sutureless vitrectomy.^5^ This is hypothesized to aid in MH closure by relieving the anteroposterior traction at the macula and creating a space for an endotamponading agent. ILM peeling and gas tamponade are believed to facilitate MH closure by removing tangential tractions.^6^ There is evidence that ILM peeling aids primary and final MH closure.^7,8^ The closure rate has been reported to be similar (>86%) with sulfur hexafluoride and perfluoropropane (C3F8) tamponade, irrespective of hole size, stage, or duration.^6^ The duration of tamponade required for hole closure has been a matter of debate. There are arguments in favor of extensive vitreous removal, although there is no way of proving that extensive vitrectomy is better than partial vitrectomy. Extensive vitrectomy means separation of the posterior hyaloid membrane up to the equator, followed by shaving of the vitreous base. The removal of as much vitreous as possible allows injection of more gas mixture into the eye and subsequently prolongs the effect of the gas tamponade.^9^

Despite new technological advancements in vitreoretinal surgery, access to the vitreous base, especially in phakic eyes, is technically challenging. Continuous infusion of balanced salt solution (BSS) is used to replace the vitreous, but air has physical properties with advantages over BSS in certain conditions. We have proposed air-infused vitrectomy with 23-gauge instrumentation for better visualization and more efficient clearing of the vitreous cavity (Frankfurt Retina Meeting; April 19-20; 2008).

The use of vitreous cutters and other instruments at the interface between perfluorocarbon liquid,^10,11,12,13^ silicone oil,^14,15,16,17^ or air,^17,18,19,20,21^ and residual vitreous, epiretinal membrane, and retina has been described as interface vitrectomy.^22^ These tamponading substances stabilize the vitreoretinal tissue because of their elevated surface tension and provide a more stable surgical field during removal of the vitreous. Vitrectomy performed under air possesses the advantage of better visualization of the peripheral vitreous and vitreous base.

Air-perfused vitrectomy has been shown to provide some benefits in cases of rhegmatogenous retinal detachment (RRD) and macular diseases.^19,20,21^ The aim of this research was to gain insight into the efficacy of air-perfused core and peripheral vitrectomy for full-thickness MH.

## Materials and Methods

A prospective, comparative study was designed. The study protocol was approved by the Ethics Committee of Şişli Memorial Hospital, İstanbul (protocol number: 0067). The study was in accordance with the principles of the Declaration of Helsinki. We enrolled 42 patients (42 eyes) with a clinical diagnosis of stage 4 idiopathic MH with complete posterior vitreous detachment who underwent small-gauge vitreoretinal surgery. The study group consisted of 21 patients who underwent vitrectomy under air infusion, and the control group of 21 patients who underwent standard vitrectomy under fluid infusion. The patients were selected randomly. Written informed consent was obtained from all participants.

All subjects underwent a comprehensive ophthalmologic evaluation, including best corrected visual acuity (BCVA), intraocular pressure, anterior segment and fundus examination, spectral-domain OCT (Spectralis; Heidelberg Engineering, Heidelberg, Germany), and microperimetry (MP1) (MP-1 Microperimeter, Nidek Technologies, Padua, Italy).

The following parameters were recorded and analyzed: demographic characteristics, preoperative and postoperative lens status, effective vitrectomy time, total surgery time, and preoperative and three-month postoperative BCVA and MP1 sensitivity.

MP1 used a red cross as the fixation target and a standardized grid of 76 Goldmann III stimuli covering the central 20°, white background illumination of 1.27 cd/m^2^, and a projection time of 200 ms, which has been described previously.^23^ A 4-2 staircase strategy was carried out and the contralateral eye was occluded during the test. All subjects underwent MP1 with dilated pupils. Differential light threshold values were compared by calculating selected points, which were averaged automatically by the MP1 software program for mean sensitivity in a polygon. MP1 (central 20°) was performed before the surgical procedure and three months postoperatively in both groups. We evaluated the difference between preoperative and postoperative MP1 results as an indirect measurement of probable adverse effect of air infusion to determine possible complications of the procedure.

All pars plana vitrectomy (PPV) procedures were performed under general anesthesia by the same surgeon (M.K.). The surgical procedures used a 3-port, transconjunctival sutureless 23-gauge vitrectomy system. In the air group, air infusion was started at the beginning of the procedure. The air pressure setting was 40 mmHg and vitrectomy was performed with a cutting rate of 2500 per min, and up to 300 mmHg of linear aspiration using the Associate 2500 vitrectomy system (DORC, Zuidland, Netherlands). The air highlighted a ring of vitreous corresponding with the vitreous base because of the air bubble pushing the vitreous residue towards the retina. The residual vitreous was removed by placing the cutter into the vitreous and shaving it until the interface profile was changed with the disappearance of the ring of vitreous as previously described.^21^ Vacuum was not applied to the vitreous cutter within the air to avoid plugging with an air lock.^22^ Scleral depression during peripheral vitrectomy was not required in the air group. At the end of the air-infused vitrectomy, an air-BSS interchange was used to remove air from the vitreous cavity. Triamcinolone acetonide-assisted peeling of the ILM was then performed circumferentially around the MH with end-gripping intraocular forceps. In case of fogging due to condensation of the lens, the posterior surface of the lens was coated with viscoelastic material.

In the standard vitrectomy group, the vitreous was removed with a cutting rate of 2500/min and up to 300 mmHg of linear aspiration up to the far periphery under fluid infusion, with dynamic scleral depression in all cases. Triamcinolone acetonide-assisted ILM peeling was performed subsequently in the same manner. Finally, surgery was completed by air-gas exchange with 12-13% C3F8 gas in both groups. None of the patients underwent vitrectomy in combination with cataract removal.

The effective vitrectomy time (from initiation of vitrectomy to the end of vitreous removal) did not include the time spent on staining and peeling the ILM.

During the postoperative period, patients were advised to maintain face-down positioning for 5-7 days. All patients were routinely examined on day one after the surgery, and at one week, one month, and three months. VA was measured with the ETDRS charts. Anatomical success was defined as apposition of the MH edges as well as the absence of any fluid cuff around the hole, as determined by clinical examination and OCT.

### Statistical Analysis

Quantitative data were expressed as means and standard deviations. The two groups were compared by using an independent-samples t-test and Mann-Whitney U test for continuous variables, and chi-square or Fisher’s exact tests for categorical variables. Wilcoxon signed rank test was used to compare the preoperative and postoperative MP1 sensitivity and VA. p*<*0.05 was considered statistically significant. Statistical *analy*ses used SPSS version 20.0 (SPSS Inc, Chicago, IL, USA).

## Results

Both groups had similar distributions of age, sex, lens status, BCVA, and retinal sensitivity. Preoperative characteristics are summarized in Table 1. The control group differed from the air group in its longer mean effective vitrectomy time and mean total surgery time (Table 2). Intraoperative complications noted during the air-infused vitrectomy were retinal touch (n=2), sudden hypotony (n=2), migration of air into the anterior chamber in a pseudophakic eye with posterior capsule defect (n=1), and fogging of the intraocular lens (n=1). Lenticular touch or lenticular opacification secondary to air infusion were not seen. In the standard vitrectomy group, no specific intraoperative complications were noted. Both groups were similar in postoperative BCVA and retinal sensitivity (Table 3). The single-procedure MH closure rate was 100% in both groups. Visually significant cataract did not develop during the three-month follow-up period.

## Discussion

We found that air-infused vitrectomy significantly reduces effective vitrectomy time and total duration of surgery. Despite some difficulties and intraoperative complications during the learning curve of this technique, it seems equally safe and comparable with standard vitrectomy technique in terms of anatomical and functional success. Although there was no significant improvement in postoperative retinal sensitivity evaluated by MP1, we concluded that continuous air infusion was not associated with significant direct or indirect damage to retinal nerve fibers.

In the standard PPV technique, vitreous gel is removed and the intraocular contents are replaced with commercially available BSS. Performing vitreoretinal surgical manipulations at the interface between perfluorocarbon liquid,^10,11,12,13^ silicone oil,^14,15,16,17^ or air,^17,18,19,20,21,22^ and residual vitreous, epiretinal membrane, and retina has been described as interface vitrectomy.^22^ In 2005, Quiroz-Mercado et al.^11^ described a technique for vitrectomy using perfluorocarbon liquids as irrigation fluids for tractional and RRD. They concluded that in selected cases the use of perfluorocarbon liquids offers several advantages over BSS, because of their properties (gravitational forces, miscibility with body fluids, and ability to transport oxygen). The main inconvenience of perfluorocarbon-infused vitrectomy is considerably higher surgical cost.^11^

Recently, Voleti et al.^18^ proposed premature fluid-air exchange so that the vitrectomy can be done under air rather than BSS. Advantages of air instead of BSS in continuous infusion during vitrectomy derive from the specific physical-mechanical properties of air. The difference in refractive index allows for a wider view of the retina under air. Moreover, the air-vitreous interface is more pronounced than the BSS-vitreous interface. Additionally, the surface tension of the air results in downward pressure over the retina and helps to further establish the interface between air and vitreous, allowing for more definitive identification of residual vitreous. This dynamic stabilizes the retina under air and, in combination with small-gauge vitreous cutters with their ports close to the tip, affords safe and precise vitrectomy. All these aspects allow easier identification of residual vitreous that should be completely removed in certain diseases (for example, RRD, proliferative diabetic retinopathy, and MH) to improve the chances of surgical success.

A widened field of view and improved visualization of both the peripheral retina and the vitreous base interface were observed in all cases during air-infused vitrectomy, allowing effective identification and removal of residual vitreous. During air-infused vitrectomy, air pushes the vitreous to the retinal surface to help prevent dispersal of the vitreous fluid. In classic BSS-infused vitrectomy, vitreous mixes with irrigating solution, and during active vitrectomy the irrigating solution is mostly aspirated. However, in the air-infused system, the vitrectomy probe stays always in the vitreous and only vitreous is aspirated. This provides more efficacious vitrectomy and lessens the effective vitrectomy time and total surgery time, as observed in our study.

Shortly after the introduction of modern PPV, air injection and fluid-air exchange gained wide acceptance at every step of vitreoretinal surgery.^24^ Fluid-air exchange during MH surgery is an essential step, required for successful closure of the hole. Visual field defects after MH surgery were reported,^25^ and vitrectomy with humidified air for fluid-air exchange was subsequently proposed to prevent these defects.^26^ Later, Gass et al.^27^ noted that peripheral visual field defects after MH surgery is a complication with decreasing incidence. They proposed that a rather low pressure setting during fluid-air exchange, as well as special aspects of the surgical technique, might be responsible for the low incidence of peripheral visual field defects. A central 20° MP1 test performed 3 months postoperatively did not indicate diffuse loss of retinal sensitivity or significant visual field defects in the air-infused vitrectomy group.

It has been reported that vitrectomy under air may play a role in reducing the rate of iatrogenic retinal break formation compared with the standard vitrectomy technique. Sigler et al.^17^ performed peripheral interface vitrectomy under air in 86 consecutive cases of RRD, none of which developed iatrogenic retinal breaks or new retinal breaks in the postoperative period. Reibaldi et al.^19^ evaluated the incidence of iatrogenic retinal breaks in small-gauge vitrectomy under air compared with standard vitrectomy for idiopathic MH or idiopathic epiretinal membranes. The incidence rates of intraoperative and postoperative retinal breaks were significantly lower in the air group (2%) than the standard group (7%). Erdogan et al.^20^ compared the efficacy and safety of peripheral vitrectomy under air infusion and fluid infusion in cases with RRD. They detected an iatrogenic retinal break rate of 2.5% in the air-infusion group, which was lower though not statistically significantly so, than the 10% in the standard vitrectomy group. In our study, with a smaller sample size, iatrogenic retinal break formation was not observed in either group.

Although the air allows a wider field of view, it can fog the posterior surface of the intraocular lens, leading to compromised posterior segment visualization. Coating the posterior lens surface with viscoelastic material will help prevent this complication. In case of intraoperative hypotony during air-infused vitrectomy, the aspiration rate should be reduced or stopped until the intraocular pressure value returns to normal.

### Study Limitations

The study was limited by the small sample size and limited follow-up.

## Conclusion

Our study showed that 23-gauge vitrectomy under air significantly reduced the effective vitrectomy time and total surgical duration compared with standard 23-gauge vitrectomy. Several intraoperative complications including retinal touch, hypotony, fogging of the intraocular lens, and air in the anterior chamber were observed in the air group. Vitrectomy under air infusion seems to be associated with several problems that may be less common in standard PPV, but a definitive conclusion is not possible given the small number of participants. The final anatomical and functional outcomes were similar for both surgical techniques. Postoperative MP1 results were not associated with loss of retinal sensitivity or significant visual field defects in the air-infused vitrectomy group, suggesting that there is no specific direct or indirect damage to the retina or optic nerve related to the continuous air infusion. Further prospective controlled studies are necessary to confirm the efficacy and safety of this surgical technique.

## Figures and Tables

**Table 1:**
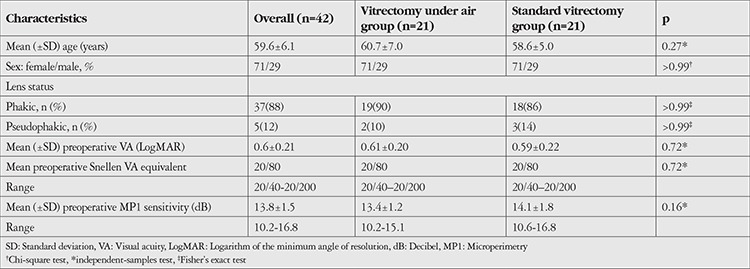
Preoperative characteristics

**Table 2:**
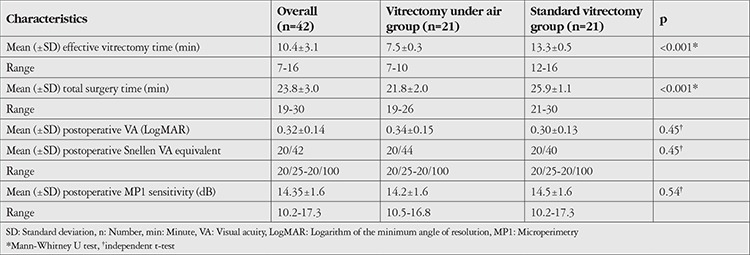
Intraoperative and postoperative outcomes

**Table 3:**
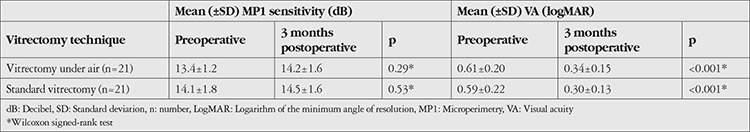
Comparisons of mean preoperative and postoperative retinal sensitivity and best corrected visual acuity
